# "Evaluating the efficacy and safety of direct oral anticoagulants compared to warfarin in very morbidly obese patients with non-valvular atrial fibrillation: A retrospective cohort study"

**DOI:** 10.1016/j.heliyon.2024.e41596

**Published:** 2025-01-03

**Authors:** Shady Ezaldin, Mahmoud Abdelsalam, Frank Annie, Julton Tomanguillo Chumbe, Elie Gharib

**Affiliations:** Cardiovascular Department, Charleston Area Medical Center, Charleston, WV, USA

## Abstract

• DOACs are effective and safe in very morbidly obese AF patients (BMI ≥50 kg/m^2^).
• DOACs show similar stroke and bleeding risks as warfarin in this population.
• Findings support DOACs in anticoagulation guidelines for very morbidly obese patients.

## 1 Introduction

In the management of nonvalvular atrial fibrillation (NVAF), the adoption of direct oral anticoagulants (DOACs) has progressively outpaced the use of warfarin for thromboembolic prevention [1,2]. Historically, the 2019 focused update by the American Heart Association (AHA), American College of Cardiology (ACC), and Heart Rhythm Society (HRS) highlighted concerns regarding DOAC use in individuals at the extremes of the body mass index (BMI) spectrum, particularly those with a BMI exceeding 35 kg/m^2^ or a weight surpassing 120 kg [3]. However, the widespread application of a uniform strategy raises concerns about the applicability, safety, and efficacy of DOACs in patients at the extremes of the body mass index (BMI) spectrum. Pharmacokinetic investigations have revealed the potential for suboptimal dosing in morbidly obese individuals and potential overdosing in underweight patients treated with DOACs [4,5].

*However, the landscape of anticoagulation in NVAF, especially for patients with high BMI, has evolved significantly with the recent* 2023 ACC*/AHA guidelines. These guidelines, informed by both meta-analyses of large DOAC trials and real-world observational studies*, have demonstrated the safety and efficacy of DOACs in patients with a BMI up to 40 kg/m^2^ [6] Furthermore, these newer guidelines show greater acceptance of DOAC use in patients with BMIs exceeding 40 kg/m^2^, recognizing that, while data remain limited, retrospective and observational studies suggest DOACs can be effective without the need for routine plasma level monitoring in many cases [6].

Despite this shift, evidence for patients with BMI >50 kg/m^2^ remains limited. In line with these updates, the 2021 European Heart Rhythm Association (EHRA) guidelines continue to advise that DOACs may be used safely and effectively in patients with a BMI up to 40 kg/m^2^, but caution is warranted for patients with a BMI over 50 kg/m^2^, where plasma level monitoring or a switch to vitamin K antagonists (VKAs) may be considered [7].

As the representation of patients with extreme BMIs remains limited in randomized trials, ongoing reliance on post-hoc analyses and real-world data is necessary to fill this gap. Pivotal DOAC trials have included a relatively small number of patients with grade 3 obesity (BMI ≥40 kg/m^2^) or underweight (BMI <18.5 kg/m^2^), leaving unanswered questions about the optimal anticoagulation strategy in these populations [[8], [9], [10], [11]].

Therefore, while recent guidelines provide more clarity on DOAC use in obese individuals, continued investigation into their safety and efficacy at the highest BMI thresholds is essential.

## 2 Methods

This retrospective cohort study utilized patient data from the TriNetX Global Collaborative Network.

### 2.1 TriNetX database description

TriNetX is a global health research network that provides real-time access to anonymized, patient data sourced from a diverse range of healthcare organizations including hospitals, academic medical centers, and primary care networks. The platform collects data from electronic medical records, billing data, disease registries, and other clinical systems to create a comprehensive dataset that can be used for clinical research and analytics.

#### 2.1.1 How TriNetX works

TriNetX's platform enables researchers to access a large patient population in real time, allowing them to identify cohorts based on specific criteria, generate hypotheses, and assess outcomes in a real-world setting. The data are de-identified at the source before being uploaded to the platform,

Data available in the platform include:

• **Demographic Information**: Age, gender, race, and geographic region.
• **Clinical Data**: Diagnoses (captured using ICD-10 codes), procedures (captured using CPT procedure codes), laboratory results, vital signs, medications, and healthcare encounters.
• **Outcomes**: Mortality, readmission, adverse events, and more.

The platform uses natural language processing and other advanced tools to extract and organize information from unstructured clinical notes, lab reports, and other documents, enhancing the richness of the dataset.

Patients included were those aged 18–90 years, diagnosed with non-valvular atrial fibrillation (AF) or atrial flutter, and with a body mass index (BMI) of at least 50 kg/m^2^. Cohorts were divided based on their prescription of warfarin (Cohort 1) or direct oral anticoagulants (DOACs: apixaban, rivaroxaban, dabigatran, or edoxaban in (Cohort 2). The ICD-10 codes used to identify atrial fibrillation and flutter included I48.0 (Paroxysmal AF), I48.1 (Persistent AF), I48.2 (Chronic AF), I48.19 (Other Persistent AF), I48.21 (Permanent AF), I48.91 (Unspecified AF), and I48.92 (Unspecified Atrial Flutter).

Exclusion criteria included conditions with strict warfarin indications or contraindications for both warfarin and DOAC and included pregnancy (ICD-10: Z34.9), the presence of a prosthetic heart valve (ICD-10: Z95.2), antiphospholipid syndrome (ICD-10: D68.61), intracardiac thrombosis (ICD-10: I51.3), rheumatic mitral valve disease (ICD-10: I05), non-rheumatic mitral valve stenosis (I 34.2). Patients with prior cerebrovascular accident (CVA) or major bleeding events were also excluded (Table 1).

**Table 1:** Cohort selection for DOAC vs warfarin study Pre and post propensity score matching.

**Criteria**	**Pre-Matching**	**Post-Matching**
**Total Patients**		
DOAC Cohort	653	377
Warfarin Cohort	384	377
**Age Range (Years)**	18–90	18–90
**Comorbidities Included**		
Non- Valvular Afib	Yes	Yes
Atrial Flutter	Yes	Yes
BMI≥ 50	Yes	Yes
**Exclusion Criteria**		
Pregnancy	Excluded	Excluded
Mechanical Heart Valves	Excluded	Excluded
Antiphospholipid syndrome	Excluded	Excluded
Left Ventricular Thrombus	Excluded	Excluded
**Comorbidities for Matching**		
Coronary Artery Disease	–	Matched
Diabetes Mellitus	–	Matched
Chronic Kidney Disease	–	Matched
Heart failure	–	Matched
Nicotine Dependence	–	Matched
Chronic Obstructive pulmonary Disease	–	Matched
History of Stroke	–	Matched
Obstructive Sleep Apnea	–	Matched

Table 1: summarizes the selection and matching criteria for a study comparing DOACs and Warfarin in morbidly obese patients. Initially, the DOAC cohort had 653 participants, and the Warfarin cohort had 384, which were balanced to 377 each through propensity score matching. Inclusion criteria were ages 18–90 with non-valvular atrial fibrillation and flutter and a BMI ≥50 kg/m^2^. Exclusions included pregnancy and certain cardiovascular conditions. Additional health issues such as coronary artery disease, smoking, CKD, diabetes among others were also considered in the matching process to ensure comparability.

Propensity score matching was used to balance the two cohorts across several variables, including age, sex, diabetes (ICD- E8-E13), chronic kidney disease (ICD-N18), chronic obstructive pulmonary disease (ICD- J44), obstructive sleep apnea (ICD-47.33), nicotine dependence (ICD-10: F17), atherosclerotic coronary artery disease (CAD) (ICD-10: I25.10) and Heart Failure (ICD-I50). The matching process aimed to minimize selection bias and ensure a balanced comparison between the warfarin and DOAC groups (Table 2).

**Table 2:** Detailed characteristics after propensity score matching.

**Characteristic**	**Warfarin Cohort (N = 377)**	**DOAC Cohort (N = 377)**	**P-Value**	**Standard Difference**
Age (Mean ± SD)	66 ± 12.1	66 ± 10.7	0.705	0.028
Female (%)	56.0 % (211)	58.9 % (222)	0.418	0.059
Male (%)	44.0 % (166)	41.1 % (155)	0.418	0.059
Atherosclerotic Heart Disease (%)	35.5 % (133)	39.3 % (148)	0.259	0.082
Nicotine Dependence (%)	39.5 % (149)	40.6 % (153)	0.766	0.022
COPD (%)	34.2 % (129)	37.9 % (143)	0.288	0.077
Diabetes Mellitus (%)	61.8 % (233)	63.1 % (238)	0.707	0.027
Chronic Kidney Disease (%)	41.4 % (156)	42.2 (163)	0.606	0.038
Obstructive Sleep Apnea (%)	49.3 % (186)	51.7 % (195)	0.512	0.048
Heart Failure (%)	56.2 % (212)	58.1 % (219)	0.606	0.038

Table 2: presents a comprehensive breakdown of the demographic and clinical profiles of the Warfarin and DOAC cohorts, each composed of 377 patients, following propensity score matching. The table compares key variables such as age, gender, and the incidence of various comorbidities including atherosclerotic heart disease, nicotine dependence, COPD, diabetes mellitus, chronic kidney disease, obstructive sleep apnea, and heart failure.

The primary outcome was the occurrence of CVAs, defined using the different ICD-10 codes. Secondary outcomes included major bleeding events including GI bleeding and CNS bleeding, as well as mortality. All the ICD-10 Codes used to identify the outcomes is included in the supplementary file A.

Outcomes were measured over 1-year and 5-year intervals, starting from the index event, which was defined as the first recorded prescription of either warfarin or a DOAC. All statistical analyses, including propensity score matching and outcome comparisons, were conducted using TriNetX's real-time analytics platform. The analysis included calculating risk ratios, odds ratios, and survival analysis using the Kaplan-Meier method.

## 3 Results

### 3.1 Cohort characteristics

After propensity score matching, our study included two well-balanced cohorts comprising 377 patients each, treated with either DOACs or warfarin. The matching process ensured comparability across key demographic and clinical variables, including age, gender, and other comorbidities associated with atrial fibrillation or atrial flutter and may affect the outcomes.

**Fig. 1:**
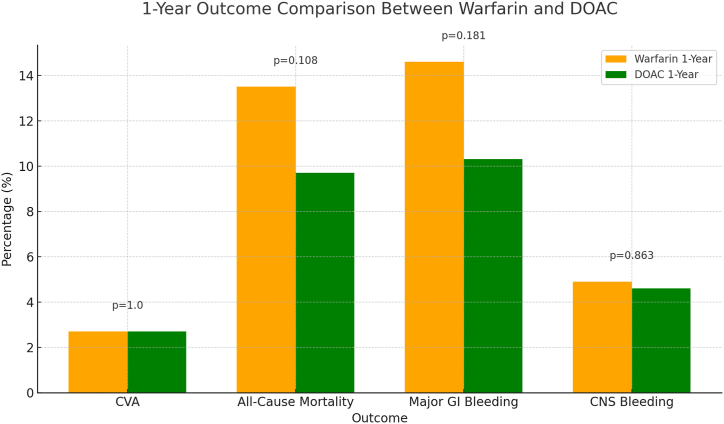
This chart displays the same outcomes as the 5-year chart but over a 1-year period.The outcomes for Warfarin are slightly higher for All-Cause Mortality and Major GI Bleeding compared to DOACs, but the percentages are generally closer between the two groups compared to the 5-year data.The p-values, ranging from 0.108 to 1.000, also indicate no statistically significant differences, suggesting that both treatments have comparable safety profiles for these outcomes within one year.
• The five-year analysis revealed similar trends, with DOACs showing slightly better outcomes over the extended follow-up period. All-cause mortality remained lower in the DOAC group (16.4 %) compared to the warfarin group (18.9 %) with p-value 0.386, and major GI bleeding was also reduced in the DOAC cohort (14.0 % vs. 16.2 %) with p-value 0.412. The rate of CVAs was marginally lower in the DOAC group (2.7 %) compared to the warfarin group (3.8 %) with p-value 0.407, while CNS bleeding was slightly more common in DOAC patients (6.2 % vs. 5.1 % for warfarin) with p-value 0.525. Although these differences did not reach statistical significance, the Kaplan-Meier survival analyses over five years supported the trend towards improved outcomes with DOACs, particularly in terms of lower mortality and fewer major bleeding events (Table 4 ,and Figs. 2 and 4).

• In the comparison of warfarin and DOACs for patients with a BMI ≥50 kg/m^2^, both the one-year and five-year analyses showed consistent trends favoring the use of DOACs over warfarin, although the results did not reach statistical significance. In the one-year analysis, the rates of CVAs were similar between the two cohorts, with both groups experiencing a 2.7 % rate of CVAs (p = 1.00). DOACs demonstrated a lower incidence of mortality (9.7 % vs. 13.5 % for warfarin) and major GI bleeding (10.8 % vs. 14.0 % for warfarin), though these differences were not statistically significant (p = 0.108 and p = 0.181, respectively). The rates of the CNS bleeding were similar between the two cohorts, with both groups experiencing 4.5–4.9 % CNS bleeding with (p = 0.863) (Table 3 ,Figs. 1 and 3).

**Table 3:** Comparison between primary and secondary outcomes between Warfarin and DOAC Cohorts over 1 year.

Outcome	Warfarin (n, %)	DOAC (n, %)	P-Value
Cerebrovascular Accidents	10 (2.7 %)	10 (2.7 %)	1.000
All-Cause Mortality	50 (13.5 %)	36 (9.7 %)	0.108
CNS Bleeding	18 (4.9 %)	17 (4.6)	0.181
Major GI bleeding	52 (14 %)	40 (10.8 %)	0.863

Table 3: illustrates a comparison of primary and secondary clinical outcomes between patients treated with Warfarin and those treated with DOACs over 1 year. The outcomes include cerebrovascular accidents, All-cause mortality, CNS bleeding, and major GI bleeding. The table displays the number and the percentages of patients experiencing each outcome in both cohorts, with corresponding p-values to assess the statistical significance of differences observed.

**Table 4:** Comparison between primary and secondary outcomes between Warfarin and DOAC Cohorts over 5 years.

Outcome	Warfarin (n, %)	DOAC (n, %)	P-Value
Cerebrovascular Accidents	14 (3.8 %)	10 (2.7 %)	0.407
All-Cause Mortality	70 (19.9 %)	61 (16.4 %)	0.386
CNS Bleeding	19 (5.1 %)	23 (6.2 %)	0.525
Major GI bleeding	60 (16.2 %)	52 (14 %)	0.412

Table 3: illustrates a comparison of primary and secondary clinical outcomes between patients treated with Warfarin and those treated with DOACs over 5 years. The outcomes include cerebrovascular accidents, All-cause mortality, CNS bleeding, and major GI bleeding. The table displays the number and the percentages of patients experiencing each outcome in both cohorts, with corresponding p-values to assess the statistical significance of differences observed.

**Fig. 2:**
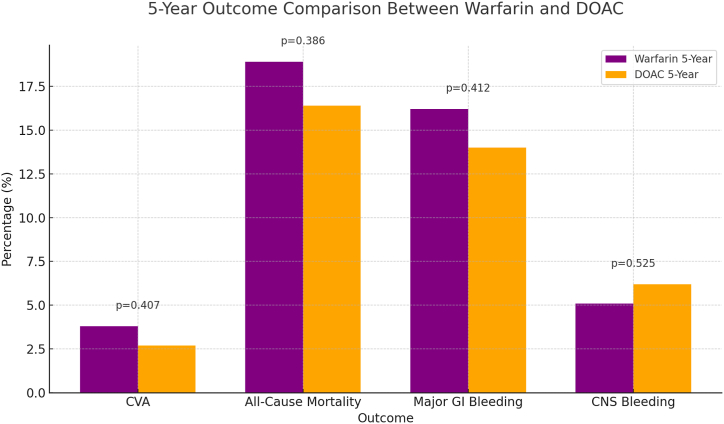
This chart compares the percentage of adverse outcomes CVAs, All-Cause Mortality, Major GI Bleeding, and CNS Bleeding between patients on Warfarin and those on DOACs over a 5-year period. Warfarin shows slightly higher percentages across most outcomes, particularly in All-Cause Mortality and Major GI Bleeding, however didn't reach statistically significant difference.

**Fig. 3:**
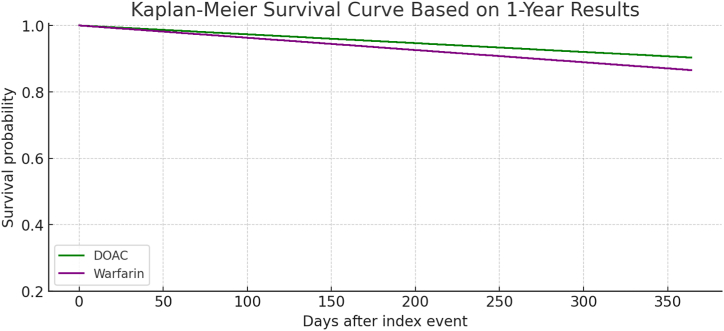
1-year Kaplan-Meier curve showed slightly higher survival for the **DOAC group** (90.3 %) compared to **warfarin** (86.5 %) in patients with a BMI ≥50 kg/m^2^, suggesting a potential short-term survival benefit with DOACs, though not statistically significant.

**Fig. 4:**
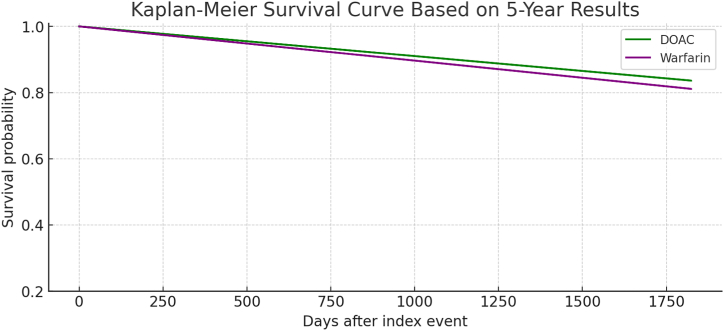
5-year Kaplan-Meier Survival Curve showed that DOAC **group** again demonstrated better survival (83.6 % vs. 81.1 % for warfarin), with a consistent trend favoring DOACs in long-term outcomes for these patients, though statistical significance was not reached.

## 4 Discussion

The safety and efficacy of DOACs compared to warfarin have been proven in the general population. Studies such as the RE-LY, ROCKET-AF, ARISTOTLE, and ENGAGE AF-TIMI 48 trials have consistently shown DOACs to be either superior or non-inferior to warfarin in preventing stroke or systemic embolism in patients with non-valvular AFib, with a comparable or lower risk of major bleeding ([[8], [9], [10], [11]] However, data on very morbidly obese patients (BMI ≥50 kg/m^2^) have been very limited, due to their underrepresentation in the pivotal trials. Our study tries to fill this gap by providing evidence that the efficacy and safety profiles of DOACs observed in the general population extend to very morbidly obese patients.

The pharmacokinetics of DOACs in obese patients is a topic of ongoing research and discussion. Data on the impact of morbid obesity (BMI ≥40 kg/m^2) on the pharmacokinetics and pharmacodynamics of DOACs are relatively limited. This limitation makes it challenging to design optimal dosing regimens for morbidly obese patients [12,13].

Obesity can affect the distribution, metabolism, and elimination of drugs, including DOACs [14]. The volume of distribution may be altered in obese patients due to changes in body composition, such as increased fat mass. Additionally, the clearance of drugs could be affected by changes in liver and kidney function associated with obesity. These factors potentially influence the plasma concentration of DOACs, their efficacy, and the risk of side effects.

Several efforts have been made to review the existing research on the pharmacokinetics/pharmacodynamics profiles, effectiveness, and safety of DOACs in treating venous thromboembolism and non-valvular atrial fibrillation in obese patients. The evidence gathered, which includes results from pivotal phase III trials, retrospective observational studies, and meta-analyses, indicates that DOACs are both effective and safe for obese patients, even those with class 3 obesity (BMI ≥40 kg/m^2). However, it's important to note that the data becomes less consistent and more variable as the BMI increases [14].

(3,6).

"In our study, we utilized data from Trinetex, . This choice was motivated by our aim to collect as large data as we can on individuals with very morbid obesity (BMI >50), a group notably underrepresented in prior clinical trials, . To ensure a good selection process, we excluded patients for whom Warfarin was indicated over DOACs due to specific clinical conditions, such as mechanical heart valve, LV thrombus, antiphospholipid syndrome, and also excluded pregnant individuals since DOACs are contraindicated in these cases as outlined in Table 1.

Our cohorts were carefully matched across different risk factors related to the primary outcome of CVAs, as detailed in Table 2. Our findings revealed no significant differences in the rates of CVAs, mortality, CNS bleeding, or major GI bleeding between the cohorts of very morbidly obese patients with non-valvular atrial fibrillation or atrial flutter, whether treated with DOACs or Warfarin. Although, we couldn't reach any significant difference between the two cohorts, that was the maximum number of patients we could obtain given the unique nature of this population –very morbidly obese patients with BMI >50- and other strict exclusion criteria.

The current landscape of using DOAC is evolving with active changes in the guidelines. Recent 2023 ACC/AHA/RHS guidelines are advocating for the use DOAC in patients with morbid obesity and that is a recent change after the cautious approach that was in the older version in 2019 (3 &6). Most of these changes are based on post-hoc anaylsis of large DOAC trials and real-world observational studies [[15], [16]]. Though there still a cautious approach for such population in some of the European guidelines as outlined in 2021 European Heart Rhythm Association with possible consideration of plasma levels measurement of switching to DOAC [7].

The results of our study can contribute to the ongoing debate regarding the optimal anticoagulation strategy in a patient population traditionally underrepresented in clinical trials and add to the evidence body for such an evolving topic. Given the logistical challenges and dietary restrictions associated with warfarin therapy, as well as the need for regular INR monitoring, DOACs represent a convenient and potentially safer alternative for very morbidly obese patients [17]. The lack of significant differences in key safety and efficacy outcomes supports the inclusion of DOACs as a viable option for anticoagulation in this population, potentially simplifying the management of AFib and atrial flutter and improving patient adherence to therapy.

### 4.1 Strengths and limitations

The utilization of a large, real-world database allowed us to include a good number of highly morbid patients who are often underrepresented or excluded in controlled studies. Furthermore, the application of propensity score matching enhanced comparability between the DOAC and Warfarin cohorts, helping to reduce potential biases.

However, as a retrospective analysis, our study is subject to inherent biases and confounding factors associated with observational data. Although propensity score matching was employed, unmeasured confounders may still affect the outcomes. Additionally, specific dosages of DOACs were not available in our analysis, which may impact both efficacy and safety results.

Another limitation is the inability to detect a statistically significant difference between the two groups. The unique characteristics of our population, combined with strict exclusion criteria, limited the cohort size, even with the extensive reach of the TriNetX database, yet the data may still offer valuable insights and contribute to the existing body of evidence.

Another limitation, we were unable to access specific laboratory information, including detailed INR monitoring and time in the therapeutic range. Consequently, we could not evaluate the percentage of patients achieving target INR for the Warfarin cohort. This limitation restricted our ability to compare INR control with DOAC users, whose therapy does not require INR monitoring. Instead, our analysis concentrated on clinical outcomes, such as cerebrovascular events, major bleeding, and mortality, as these data were consistently available across both cohorts.

Lastly, outcome validation was limited by TriNetX's reliance on ICD-10 coding, which depends on the accuracy of the coding at the time of diagnosis. While we used comprehensive ICD-10 codes to capture outcomes, the reliability of our findings ultimately depends on the accuracy of these codes.

### 4.2 Future research

More research is needed to explore the impact of different DOAC dosages and regimens in very morbidly obese patients and to investigate the long-term outcomes of anticoagulation therapy in this population. Randomized controlled trials that specifically include very morbidly obese patients are necessary to definitively establish the role of DOACs compared to warfarin in managing AFib and atrial flutter within this demographic.

## 5 Conclusion

In conclusion, our study suggests that DOACs are a safe and effective alternative to warfarin for anticoagulation in very morbidly obese patients with non-valvular AFib or atrial flutter. These findings support the broader application of DOACs across various patient populations, including those with significant obesity, thereby informing clinical decision-making and guideline development. Further research is essential to fully understand the use of anticoagulation therapy in this unique patient population.

## CRediT authorship contribution statement

**Shady Ezaldin:** Writing – original draft, Visualization, Project administration, Methodology, Investigation, Conceptualization. **Mahmoud Abdelsalam:** Writing – review & editing, Investigation, Conceptualization. **Frank Annie:** Writing – review & editing, Supervision, Software, Formal analysis, Data curation. **Julton Tomanguillo Chumbe:** Software, Methodology, Formal analysis, Data curation. **Elie Gharib:** Writing – review & editing, Supervision, Methodology, Conceptualization.

## Statement

During the preparation of this work the authors used ChatGPT to review vocabulary and enhance readability. After using this tool, the authors reviewed and edited the content as needed and take full responsibility for the content of the publication.

## Declaration of competing interest

The authors declare that they have no known competing financial interests or personal relationships that could have appeared to influence the work reported in this paper.
