# Supplementary material for: "Evaluating the efficacy and safety of direct oral anticoagulants compared to warfarin in very morbidly obese patients with non-valvular atrial fibrillation: A retrospective cohort study"
Source: Heliyon. 2025 Jan 3;11(1):e41596. doi: 10.1016/j.heliyon.2024.e41596 (PMC11758958; doi:10.1016/j.heliyon.2024.e41596)
Supplement: Multimedia component 1 [file mmc1.docx]

This File contain all the ICD-10 codes that were used for inclusion, exclusion, matching and outcomes

ICD Codes for Atrial Fibrillation and Atrial flutter

1. **Atrial Fibrillation and Atrial Flutter Codes**:
   1. **I48.0**: Paroxysmal atrial fibrillation
   2. **I48.1**: Persistent atrial fibrillation
   3. **I48.2**: Chronic atrial fibrillation
   4. **I48.19**: Other persistent atrial fibrillation
   5. **I48.21**: Permanent atrial fibrillation
   6. **I48**: Atrial fibrillation and flutter (general category)
   7. **I48.20**: Chronic atrial fibrillation, unspecified
   8. **I48.11**: Longstanding persistent atrial fibrillation
   9. **I48.91**: Unspecified atrial fibrillation
   10. **I48.92**: Unspecified atrial flutter
   11. **I48.3**: Typical atrial flutter
   12. **I48.4**: Atypical atrial flutter
   13. **I48.9**: Unspecified atrial fibrillation and atrial flutter

The ICD-10 codes used for **exclusion criteria**:

1. **Z34.9**: Encounter for Pregnancy
2. **Z95.2**: Presence of prosthetic heart valve
3. **D68.61**: Antiphospholipid syndrome
4. **I51.3**: Intracardiac thrombosis.
5. **I05**: Rheumatic mitral valve diseases

The ICD-10 codes used for **matching criteria:**

1. **I25.1**: Atherosclerotic heart disease of native coronary artery
2. **Z87.891**: Personal history of nicotine dependence
3. **J44**: Other chronic obstructive pulmonary disease
4. **E08-E13**: Diabetes mellitus
5. **N18**: Chronic kidney disease
6. **G47.33**: Obstructive sleep apnea
7. **I50**: Heart failure

The ICD-10 codes related to **Cerebrovascular Accident (CVA)** or **Cerebral Infarction:**

1. **Cerebral Infarction due to Thrombosis and Embolism**:
   1. **I63.0**: Cerebral infarction due to thrombosis of precerebral arteries
   2. **I63.1**: Cerebral infarction due to embolism of precerebral arteries
   3. **I63.2**: Cerebral infarction due to unspecified occlusion or stenosis of precerebral arteries
   4. **I63.3**: Cerebral infarction due to thrombosis of cerebral arteries
   5. **I63.4**: Cerebral infarction due to embolism of cerebral arteries
2. **Cerebral Infarction due to Embolism**:
   1. **I63.12**: Cerebral infarction due to embolism of basilar artery
   2. **I63.11**: Cerebral infarction due to embolism of vertebral artery
   3. **I63.10**: Cerebral infarction due to embolism of unspecified precerebral artery
   4. **I63.19**: Cerebral infarction due to embolism of other precerebral artery
   5. **I63.13**: Cerebral infarction due to embolism of carotid artery
3. **Cerebral Infarction due to Thrombosis in Specific Cerebral Arteries**:
   1. **I63.30**: Cerebral infarction due to thrombosis of unspecified cerebral artery
   2. **I63.31**: Cerebral infarction due to thrombosis of middle cerebral artery
   3. **I63.33**: Cerebral infarction due to thrombosis of posterior cerebral artery
   4. **I63.34**: Cerebral infarction due to thrombosis of cerebellar artery
4. **Cerebral Infarction due to Embolism in Specific Cerebral Arteries**:
   1. **I63.40**: Cerebral infarction due to embolism of unspecified cerebral artery
   2. **I63.41**: Cerebral infarction due to embolism of middle cerebral artery
   3. **I63.43**: Cerebral infarction due to embolism of posterior cerebral artery
   4. **I63.44**: Cerebral infarction due to embolism of cerebellar artery
5. **Cerebral Infarction due to Unspecified Occlusion or Stenosis of Cerebral Arteries**:
   1. **I63.5**: Cerebral infarction due to unspecified occlusion or stenosis of cerebral arteries
   2. **I63.50**: Unspecified cerebral artery
   3. **I63.51**: Middle cerebral artery
   4. **I63.52**: Anterior cerebral artery
   5. **I63.53**: Posterior cerebral artery
   6. **I63.54**: Cerebellar artery
6. **Other Specific Embolism or Thrombosis Sites in Cerebral and Precerebral Arteries**:
   1. Includes codes for specific vessels such as **I63.42, I63.419, I63.413, I63.411, I63.412**, among others.

The list of **ICD-10 codes related to CNS Bleeding** included in your document:

1. **Nontraumatic Subarachnoid Hemorrhage**:
   1. **I60.00**: Nontraumatic subarachnoid hemorrhage from unspecified carotid siphon and bifurcation
   2. **I60.01**: Nontraumatic subarachnoid hemorrhage from right carotid siphon and bifurcation
   3. **I60.02**: Nontraumatic subarachnoid hemorrhage from left carotid siphon and bifurcation
   4. **I60.10**: Nontraumatic subarachnoid hemorrhage from unspecified middle cerebral artery
   5. **I60.11**: Nontraumatic subarachnoid hemorrhage from right middle cerebral artery
   6. **I60.12**: Nontraumatic subarachnoid hemorrhage from left middle cerebral artery
   7. **I60.2**: Nontraumatic subarachnoid hemorrhage from anterior communicating artery
   8. **I60.31**: Nontraumatic subarachnoid hemorrhage from right posterior communicating artery
   9. **I60.30**: Nontraumatic subarachnoid hemorrhage from unspecified posterior communicating artery
   10. **I60.32**: Nontraumatic subarachnoid hemorrhage from left posterior communicating artery
   11. **I60.4**: Nontraumatic subarachnoid hemorrhage from basilar artery
   12. **I60.50**: Nontraumatic subarachnoid hemorrhage from unspecified vertebral artery
   13. **I60.51**: Nontraumatic subarachnoid hemorrhage from right vertebral artery
   14. **I60.52**: Nontraumatic subarachnoid hemorrhage from left vertebral artery
   15. **I60.6**: Nontraumatic subarachnoid hemorrhage from other intracranial arteries
   16. **I60.7**: Nontraumatic subarachnoid hemorrhage from unspecified intracranial artery
   17. **I60.8**: Other nontraumatic subarachnoid hemorrhage
   18. **I60.9**: Nontraumatic subarachnoid hemorrhage, unspecified
2. **Nontraumatic Intracerebral Hemorrhage**:
   1. **I61.0**: Nontraumatic intracerebral hemorrhage in hemisphere, subcortical
   2. **I61.1**: Nontraumatic intracerebral hemorrhage in hemisphere, cortical
   3. **I61.2**: Nontraumatic intracerebral hemorrhage in hemisphere, unspecified
   4. **I61.3**: Nontraumatic intracerebral hemorrhage in brain stem
   5. **I61.4**: Nontraumatic intracerebral hemorrhage in cerebellum
   6. **I61.5**: Nontraumatic intracerebral hemorrhage, intraventricular
   7. **I61.6**: Nontraumatic intracerebral hemorrhage, multiple localized
   8. **I61.8**: Other nontraumatic intracerebral hemorrhage
   9. **I61.9**: Nontraumatic intracerebral hemorrhage, unspecified
3. **Nontraumatic Subdural and Extradural Hemorrhage**:
   1. **I62.00**: Nontraumatic subdural hemorrhage, unspecified
   2. **I62.01**: Nontraumatic acute subdural hemorrhage
   3. **I62.02**: Nontraumatic subacute subdural hemorrhage
   4. **I62.03**: Nontraumatic chronic subdural hemorrhage
   5. **I62.1**: Nontraumatic extradural hemorrhage
   6. **I62.9**: Nontraumatic intracranial hemorrhage, unspecified

The **ICD-10 codes related to Major Gastrointestinal Bleeding** :

1. **Esophageal Varices with Bleeding**:
   1. **I85.01**: Esophageal varices with bleeding
   2. **I85.11**: Secondary esophageal varices with bleeding
2. **Esophageal Ulcer with Bleeding**:
   1. **K22.11**: Ulcer of esophagus with bleeding
3. **Gastric Ulcer with Bleeding**:
   1. **K25.0**: Acute gastric ulcer with hemorrhage
   2. **K25.2**: Acute gastric ulcer with both hemorrhage and perforation
   3. **K25.4**: Chronic or unspecified gastric ulcer with hemorrhage
   4. **K25.6**: Chronic or unspecified gastric ulcer with both hemorrhage and perforation
4. **Duodenal Ulcer with Bleeding**:
   1. **K26.0**: Acute duodenal ulcer with hemorrhage
   2. **K26.2**: Acute duodenal ulcer with both hemorrhage and perforation
   3. **K26.4**: Chronic or unspecified duodenal ulcer with hemorrhage
5. **Peptic Ulcer with Bleeding (Unspecified Site)**:
   1. **K27.2**: Acute peptic ulcer, site unspecified, with both hemorrhage and perforation
   2. **K27.4**: Chronic or unspecified peptic ulcer, site unspecified, with hemorrhage
   3. **K27.6**: Chronic or unspecified peptic ulcer, site unspecified, with both hemorrhage and perforation
6. **Gastrojejunal Ulcer with Bleeding**:
   1. **K28.0**: Acute gastrojejunal ulcer with hemorrhage
   2. **K28.4**: Chronic or unspecified gastrojejunal ulcer with hemorrhage
   3. **K28.6**: Chronic or unspecified gastrojejunal ulcer with both hemorrhage and perforation
7. **Gastritis with Bleeding**:
   1. **K29.01**: Acute gastritis with bleeding
   2. **K29.21**: Alcoholic gastritis with bleeding
   3. **K29.31**: Chronic superficial gastritis with bleeding
   4. **K29.41**: Chronic atrophic gastritis with bleeding
   5. **K29.51**: Unspecified chronic gastritis with bleeding
   6. **K29.61**: Other gastritis with bleeding
   7. **K29.71**: Gastritis, unspecified, with bleeding
8. **Duodenitis and Gastroduodenitis with Bleeding**:
   1. **K29.81**: Duodenitis with bleeding
   2. **K29.91**: Gastroduodenitis, unspecified, with bleeding
9. **Other GI Bleeding Conditions**:
   1. **K31.811**: Angiodysplasia of stomach and duodenum with bleeding
   2. **K31.82**: Dieulafoy lesion (hemorrhagic) of stomach and duodenum
   3. **K55.21**: Angiodysplasia of colon with hemorrhage
10. **Diverticulosis and Diverticulitis with Bleeding**:

- **K57.01**: Diverticulitis of small intestine with perforation and abscess with bleeding
- **K57.11**: Diverticulosis of small intestine without perforation or abscess with bleeding
- **K57.13**: Diverticulitis of small intestine without perforation or abscess with bleeding
- **K57.21**: Diverticulitis of large intestine with perforation and abscess with bleeding
- **K57.33**: Diverticulitis of large intestine without perforation or abscess with bleeding
- **K57.41**: Diverticulitis of both small and large intestine with perforation and abscess with bleeding
- **K57.51**: Diverticulosis of both small and large intestine without perforation or abscess with bleeding
- **K57.53**: Diverticulitis of both small and large intestine without perforation or abscess with bleeding
- **K57.81**: Diverticulitis of intestine, part unspecified, with perforation and abscess with bleeding
- **K57.91**: Diverticulosis of intestine, part unspecified, without perforation or abscess with bleeding
- **K57.93**: Diverticulitis of intestine, part unspecified, without perforation or abscess with bleeding

1. **Other GI Bleeding Sites**:

- **K62.5**: Hemorrhage of anus and rectum
- **K63.81**: Dieulafoy lesion of intestine
- **K66.1**: Hemoperitoneum
- **K92.2**: Gastrointestinal hemorrhage, unspecified
